# Teachers’ understandings of the social and professional support needed to implement change in Qatar

**DOI:** 10.1016/j.heliyon.2022.e08818

**Published:** 2022-01-22

**Authors:** Ibrahim M. Karkouti, Reem Khalid Abu-Shawish, Michael H. Romanowski

**Affiliations:** a Graduate School of Education, The American University in Cairo, Cairo, Egypt; b College of Education, Qatar University, Doha, Qatar; c Educational Research Center, College of Education, Qatar University, Doha, Qatar

**Keywords:** Educational change, Social support, Teacher development, Teacher stress

## Abstract

Currently, Qatar is implementing an educational reform to enhance teaching and learning in public and private schools. The Qatar National School Accreditation significantly impacted Qatar's private schools, requiring teachers to implement various mandated educational changes. Using House (1981) types of social support, this qualitative, phenomenological study was designed to explore teachers' understandings regarding the social and professional support they need to implement educational change at an international school in Qatar. To help teachers engage in change, findings revealed that educational leaders need to heed teacher wellbeing during educational reform, educational change should be contextualized and tailored to the needs of teachers, and support should be offered to reduce teachers' stress and facilitate the change process. Recommendations for educational leaders trying to help teachers implement mandated educational change are provided in light of the derived findings.

## 1 Introduction

Worldwide, the globalization of educational services has shaped educational policy and reforms by inundating nations with transferred educational policies and practices that provide countries with opportunities to repair or transform their education systems (Romanowski and Du, 2020; Oplatka, 2018). Policymakers mistakenly believe that educational change occurs by simply initiating or reconstructing school structures and that adopted educational reforms are quite complicated, requiring significant changes (Cuban, 2013; Portnoi, 2016). The result is government-imposed top-down approaches that do not consider teachers' wellbeing during reform implementation (Arar et al., 2019; Portnoi, 2016). The realization of educational reform is determined by principals’ and teachers' acceptance, involvement, and degree of ownership of the reform (Arar et al., 2019). More importantly, top-down reform approaches prove ineffective because they fail to recognize the need for buy-in from educators (Portnoi, 2016). When teachers are required to implement change, supporting teachers becomes critical (Gibson and Brooks, 2012; Melville et al., 2012).

In Qatar, a member state of the Gulf Cooperation Council (GCC), which is an intergovernmental political union that includes Bahrain, Kuwait, Oman, Saudi Arabia and United Arab Emirates, principals have been struggling to enlist their teachers in reform initiatives due to a lack of social and professional support. This is also the case of education reform in other GCC states that have developed reforms to overhaul their educational systems (Gonzalez et al., 2008). For example, and relevant to this paper, Qatar's reform, *Education for a New Era* established in 2002 has experienced numerous alterations from its original design, leaving a long list of policies, strategies, and programs implemented to provide schools with the possibility of improving education in government (public schools) and private schools who receive minimal financial support from public entities and solely rely on the payment of fees which parents pay to have their children go to. However, amid a gradual re-centralization of Qatar's education, the recommendations regarding standards-based systems have remained a cornerstone of educational policy and programs leading to the current Qatar National School Accreditation (QNSA). The QNSA, an adopted national accreditation system governed by the Ministry of Education and Higher Education (MEHE), will be developed here because it is most relevant for this study since QNSA has a significant impact on Qatar's private schools. The vision for QNSA states:

To build a national accreditation system matching global accreditation systems in performance and quality. QNSA aims to achieve the principles of the education system in the State of Qatar in an era of educational reform, ensuring that schools are continuously and consistently improving standards through self-evaluation and action planning. (MEHE, 2017, para. 1)

Specifically, the MEHE requires private and public schools to engage in self-assessment and improvement plans by comparing their performance and learning outcomes against the standards set forth by QNSA (QNSA, 2017). Failure to do so will lead to the revocation of their national accreditation, which in turn jeopardizes the school mission. Certainly, all of this directly impacts teachers.

This study is significant for several reasons. First, it adds to the literature available on social support in K-12 schools in the Arab world, specifically Qatar. Although research has been conducted in many countries regarding the effects of social and professional support on K-12 education (e.g., Fernández-Lasarte et al., 2020; Chen et al., 2020), few, if any, studied private school teachers in Qatar. With that in mind, this study uses House (1981) social support framework to explore the pragmatic aspects of educational change. Specifically, the study explores private school teachers' understanding of social and professional support they need to implement educational change in Qatar.

## 2 Qatar's educational system and reform

Since 2003, Qatar has been involved in an ongoing educational reform that has reshaped the educational landscape. This reform implemented a decentralized approach to education only later returning to a recentralized system (Romanowski and Du, 2020). For this research, one essential element of Qatar's reform was "no matter what else was to occur, the basic educational elements of a standards-based system had to be put in place" (Brewer et al., 2007, p. xviii). This has led to the borrowing and implementing curriculum standards, professional standards for school leaders and teachers, and Qatar's National School Accreditation (For a detailed account of Qatar educational reform, see Nasser, 2017).

Since the MEHE operates in a centralized system, it has full authority and power to implement school change. Principals and teachers in Qatar face a barrage of top-down new educational policies and practices they are required to implement (Romanowski et al., 2019; Nasser, 2017). However, policymakers in Qatar often disregard the importance of engaging teachers in policy and decision-making and set unrealistic expectations, create burdening workloads and ambiguous guidelines, all in the name of change (Romanowski et al., 2013; Zellman et al., 2009). These include but are not limited to national curriculum standards, professional standards for teachers and leaders, proficiency testing, and other requirements for QNSA (Nasser, 2017).

Regarding the QNSA, the MEHE believes that school accreditation is imperative for schools to assess the quality of the education provided “through a critical self-study; and development and monitoring of a school improvement plan” (MEHE, 2017, para. 2). QNSA requires teachers to shift teacher-centered instruction to student-centered learning that focuses on differentiated instruction and integrating technology into teaching and learning. Moreover, teachers are responsible for: (a) developing appropriate assessment methods to monitor student progress and measure their learning outcomes, (b) allocating resources, and (c) modifying curricula to fit students' needs. Given QNSA's stipulations in Qatar's context, teachers need to respond to professional teaching standards by abandoning outdated teaching methods that emphasize rote memorization and applying modern teaching techniques such as differentiated learning, metacognitive methods, and critical thinking strategies.

## 3 Literature review

### 3.1 Teachers’ response to education reform

Educational reform requires teachers to adapt or change their belief systems and their educational practices during the implementation process (McDonald, 2012). Tabari (2014) contends that a vital element of this change is teachers' involvement and reaction to change because this plays a significant role in determining the implementation and effectiveness of educational reforms. Concerning mandated change, Naraian and Schlessinger (2018) examined reform in the United States and demonstrated that teachers reported a lack of support during education change and with the endeavor itself. While the most crucial factor in improving teachers' reactions toward change was the involvement of teachers in the process, teachers indicated a negative view of the reforms' mandated nature. Similarly, Tabari (2014) studied Emirati teachers and non-Emirati teachers with Arab nationality in the United Arab Emirates regarding their educational reform beliefs. Findings demonstrated that teachers were generally supportive of the development of the country's vision for education. However, resistance stemmed from the fast-paced change and large-scale reform. Teachers reported that they were not given enough resources, adequate support, and sufficient time to acquaint themselves with the changes making it extremely challenging for implementation (Tabari, 2014).

In Qatar, top-down educational changes or policies imposed upon teachers often fall short due to teachers' lack of enthusiasm for innovation and change (Alfadala, 2015). Although top-down reforms possibly increase implementation efficiency, teachers who implement the reform struggle due to a lack of understanding, motivation, and skills and become obstacles to achieving the reform's goals, making the reform less effective (Al Said et al., 2019). Top-down reform often fails to consider teachers' input and social needs, which silences teachers' voices and hampers reform because mandated reforms can be an intimidating experience producing fear, anxiety, and a sense of powerlessness for teachers (Craig, 2020).

### 3.2 Teachers’ need for support

To improve education systems, GCC countries often engage in educational borrowing by implementing ideas that have been successful in other countries hoping these will be quick fixes that deliver fast results (Romanowski and Du, 2020; Donn and Al Manthri, 2013). These products include teacher training programs, national professional standards, and school accreditation bodies or systems. There is no shortage of criticism of educational borrowing (e.g., Abu-Shawish et al., 2021; Nguyen-Phuong-Mai et al., 2012; Steiner-Khamsi, 2016). A complete discussion of these is for another venue.

Oplatka (2018) argues that educational reform is complex and that the implementation of educational reforms results in changing the status quo, propelling teachers' enthusiasm to change. Despite the possible disadvantages of borrowed reforms, teachers are asked to adapt or change their belief systems and educational practices to implement mandated change (Romanowski and Du, 2020), which is Qatar's case. Research suggests that teachers are more likely to report less stress, better job satisfaction, and health outcomes when their administration supports their decisions inside the classroom and when principals provide them with feedback regarding their work (Wright, 2017). All this suggests that school leaders consider their in-school reform efforts and purposely support the complex needs of teachers (Lockton and Fargason, 2019).

## 4 Theoretical framework

Since Qatar is amid educational reform, this qualitative, phenomenological study explores the types of social and professional support teachers need to implement educational change effectively. Neubauer et al. (2019) contend that phenomenological research assists researchers as they interpret and explain complex phenomena. Using qualitative interview data to explore a confined system in depth (a single school) (Clark and Creswell, 2014), phenomenology was selected for this study. Furthermore, the purpose of phenomenological research is to provide in-depth explanations of phenomena from the participants' perspectives (Willis, 2007). Therefore, the researchers chose a theoretical framework designed by House (1981) to explore the pragmatic aspects of educational change through teachers' eyes experiencing that change. The researchers used House (1981) comprehensive framework, which views all possible social and professional support as an individual's perception of the support they receive in their context that enhances functioning or creates adverse outcomes (Malecki and Demaray, 2002). House's framework clarifies types of social support, sources of social support, the nature of individuals who receive it, and the context of problems that needs it. More specifically, it seeks to answer the question: “Who gives what to whom regarding which problems?” (House, 1981, p. 22).

According to House (1981), social support content can be categorized into four types: emotional, informational, instrumental, and appraisal. Social support is associated with how people cope with stressful events; in this context, teachers dealing with educational reform. Table 1 contains a brief description of each.

**Table 1:** Forms of social support (House, 1981).

Types of Social Support	Description
Emotional	Sharing life experiences and empathizing with individuals to help them overcome job-related stress.
Informational	Devising plans and suggesting actions that aid in problem-solving.
Instrumental	Providing tangible support or direct assistance to help individuals achieve their goals.
Appraisal	Promoting a culture of self-evaluation, constructive feedback, and affirmation.

## 5 Research questions

This study explores teachers’ understanding of their social and professional support to engage in educational change. Teachers were only asked to describe and discuss their perspectives regarding the challenges they face during educational change and the types of support they need. Specifically, the research questions that guided this study are:

1. What are the challenges teachers faces during educational change?
2. What are teachers' understandings regarding the social support they need when implementing educational change?

## 6 Method

To understand teachers' experiences in their need for social and professional support in their daily practices, this study used a phenomenological approach to reflect upon and describe their experience through interactions with the interviewer (Van Manen, 2014). This method's strength is the ability to provide a comprehensive description of individuals' experiences that they live through in daily practice to better understanding the topic being examined (O'Brien et al., 2017; Van Manen, 2014). The purpose is to accurately portray these teachers to describe what exists and possibly discover new meanings.

### 6.1 Participants and setting

Private schools are often named international or community schools in Qatar, but they are still private non-government schools. As of spring 2019, 543 private schools are operating in Qatar, providing education for 62% of Qatar's students (Pathak, 2019). The New International School (NIS)^1^, where this study was conducted, is a private K-12 mixed gender school that enrolls 1053 students (56.98% female; 43.02% male) of diverse nationalities and backgrounds. This site was selected based on convenience in that the researchers could gain access and cooperation from the administration and faculty. The school's instructional staff consists of 103 teachers from neighboring Arab countries, primarily Egypt, Lebanon, and Jordan. None of the employed teachers are Qatari nationals. The student-teacher ratio is 10.2:1. English is the primary language of instruction, and the classroom contact hour is 45 min with the typical teaching load of five or six classes per day. To hone their teaching skills, the school administration conducts workshops and professional development for all teachers weekly. Also, the school offers mentorship opportunities for new faculty during their first teaching year at school. New teachers usually get lower teaching loads in their first semester to help them acculturate and adapt to the new system.

The principal decided that teachers in the school could participate, which is Qatar's standard procedure for research studies. All teachers in the schools were invited to participate. Thus, all participants were volunteers. The sample for the study consisted of 20 teachers (65% female; 35% male) from NIS who are implementing various educational changes directed by the MEHE, the governing body for all education in the country. Table 2 provides an overview of the participating teachers.

**Table 2:** Overview of participating teachers.

Pseudonyms	Gender	Degree Awarding Nation	Years at NIS	Teaching Area	Fluency in English
Alia	F	Lebanon	14	English	Fluent
Samer	M	Lebanon	14	Math	Fluent
Rana	F	Canada	10	Science	Fluent
Adam	M	USA	8	Math	Fluent
Mira	F	Lebanon	7	English	Fluent
Ayman	M	Lebanon	8	Science	Fluent
Yomna	F	USA	9	English	Fluent
Omar	M	Qatar	10	Math	Fluent
Mariam	F	Qatar	10	Social Studies	Fluent
Farah	F	Egypt	10	Science	Fluent
Sami	M	Lebanon	11	Physics	Fluent
Marwa	F	Egypt	13	Chemistry	Fluent
Samar	F	Jordan	14	Biology	Fluent
Daniel	M	Lebanon	6	Biology	Fluent
Rasha	F	Syria	10	Economics	Moderately Fluent
Sarah	F	Kuwait	8	Math	Moderately Fluent
Tania	F	Lebanon	7	Accounting	Fluent
Salam	M	Lebanon	11	Physics	Fluent
Mirna	F	USA	12	Chemistry	Fluent
Jaida	F	Lebanon	12	English	Fluent

### 6.2 The interviews

The purpose of the interviews was to explore in-depth participants' lived experiences working to meet QNSA accreditation. Semi-structured interviews were conducted in either English or Arabic, depending on the informant's preference. The first author, who is bilingual (Arabic/English), met individually with each participant at their convenience to explain the study procedures. The researcher informed the participants that they could withdraw during the study and that they had the option not to respond to any question. Before conducting the interviews, each participant signed a consent form and completed a demographics sheet that helped the researchers identify relevant demographic factors. This sheet asked participants to provide their gender, nationality, and years of classroom teaching experience. To answer the research questions, the study participants answered six interview questions (see AppendixA).

Probing was used throughout the interviews to develop teachers' responses further and develop relevant examples. Each interview was audio-recorded and later transcribed and translated into verbatim transcripts to present the findings in a narrative form. Also, the researcher made handwritten notes to track valuable insights that could enrich the findings. To validate the results, each participant was provided with an interview transcript for final revision. The interviews occurred at the school and lasted approximately 45–50 min. Finally, to ensure confidentiality and identity protection, each of the teachers is identified with a pseudonym.

### 6.3 Data analysis

The objective of qualitative data analysis is to develop complex meanings that emerge from raw data. Content analysis was the approach used for data analysis (Bryman, 2012). Interviews were transcribed and then Arabic interviews were translated to English to enable all researchers to analyze the data. Using the research questions as a guide, the data was analyzed using Clark and Creswell's (2014) three stages, exploring, coding, and refining and building findings. Direct content analysis where codes are developed prior and during the analysis was used (Hsieh and Shannon, 2005). The findings were compared, and upon researcher consensus, data were organized in ways that facilitated the identification of meaningful themes and patterns based on the four forms of social support, and relevant quotes were integrated into various themes. This consensus allowed for triangulation to check codes, refine emergent themes, and evaluate the study findings.

### 6.4 Trustworthiness

To establish rigor in qualitative research, the researchers implemented Tracy's (2010) eight-dimension model for quality in qualitative research: (a) worthy topic, (b) rich rigor, (c) sincerity, (d) credibility, (e) resonance, (f) significant contribution, (g) ethics, and (h) meaningful coherence. In addition, the researchers applied multiple strategies to enhance credibility, transferability, dependability, and confirmability of the results. First, member checking enabled the participants to double check their responses before data analysis (Creswell, 2012). Once data analysis was complete, each of the participants reviewed the interpreted findings for data validation and verification purposes. The end goal was to provide an accurate account of the participants' understandings regarding the social support needed at one particular international school in Qatar. Second, in order to enhance both transferability and dependability, the researchers carefully reported data collection and analysis procedures, cross-checked emergent themes to avoid redundancy and data overlapping before presenting them as narrative paragraphs, and reviewed the study protocol to allow research replication (Clark and Creswell, 2014). Finally, to avoid bias in research, the researchers who are aware of the research setting used a journal log to reflect on their thoughts while conducting the study. In turn, this helped them eliminate subjectivity, maintain objectivity, and sustain neutrality during data collection and analysis.

## 7 Limitations

There were several limitations to this research. First, this is an initial study that explores private school teachers' understandings of the social and professional support they need to implement change. Findings are not conclusive since the study was designed to explore the topic simply. Results from this study could serve as a basis for additional research to construct a research design or to develop a questionnaire to survey a large sample. Second, self-reported data in this study could suggest that some responses may lack some accuracy when probing a particular issue because some participants may be hesitant to portray a negative perspective of their school. Besides, the relatively low number of participants (*N* = *20*) might be considered a limitation. Third, it would be worthwhile for future studies to triangulate these findings by examining administrators' perspectives regarding the social and professional support teachers need and their support as school leaders. Replicating this study by including multiple research sites, sampling a broader population, including administrators, and using multiple data collection tools could provide valuable insights. Furthermore, this study does not center on Qatar's cultural context's values, norms, and other aspects that could shape how these teachers understand social support and how these factors could influence the social support they might need. Instead, the study provides valuable insight into private school teachers' perspectives regarding the social support to implement change.

## 8 Findings

The results of the study are presented in five sections. The first section answers the first two interview questions and summarizes teachers' understandings regarding the most challenging and stressful changes when implementing top-down changes from QNSA. Then, the remainder of the interview questions is addressed and different forms of social and professional support are reported. Each section centers on the major themes that emerged from the datasets. Finally, the discussion part addresses teachers’ perceptions of the types of social support they need the most in order to implement educational change at school and draws connection between study findings and the available literature on the topic.

### 8.1 Perceived job stresses

According to the participants, teachers face several changes that lead to a stressful work environment. The greatest challenge for these teachers centered upon the need for changes or improvements in their teaching practices. In particular, integrating technology into their teaching, redesigning curriculum, applying new lesson plan formats, and implementing differentiated instruction in their lessons were significant concerns for those teachers. Table 3 breaks down teachers’ understanding of the most stressful requirements that impede their educational change efforts.

**Table 3:** Teachers’ understandings of the stressful change demands required by the MEHE.

Perceived Stressful Changes	Number of Respondents	%
Technology Integration	3	15%
Redesigning Curriculum	7	35%
Lesson Planning	5	25%
Differentiated Instruction	11	55%

#### 8.1.1 Technology integration

Three teachers reported the integration of technology into their teaching as a stressful change requirement. This issue was twofold. First, some teachers seemed to lack the skills needed to integrate technology into their teaching effectively. In support of this argument, Alia reported, “Today, we are required to use technology inside our classrooms, but we are not trained on using Smart Boards or interactive projectors." Second, since technology integration is among the MEHE educational reform requirements, teachers have become increasingly frustrated with this changing demand due to the poor technological environment. Teachers suggested that classes are not equipped with technology resources, making the MEHE requirement extremely difficult.

#### 8.1.2 Redesigning curriculum

Roughly one-third of those teachers suggested redesigning and aligning the curriculum to the national standards as a stressful change that the MEHE educational reform entails. This is partly due to teachers' skills, competencies, and areas of expertise that do not support instant drastic changes. In support of this argument, Rana explained, "The hardest thing I am facing is redesigning my curriculum. I do not know what learning objectives I should omit or add. This is so confusing because designing a curriculum is not my area of expertise."

#### 8.1.3 Lesson planning

The newly proposed lesson plan formats represented a significant concern for five teachers who explained that structured lesson planning does not help them apply the required changes. The respondents argued that the new lesson plan templates are ineffective and time-consuming. For example, Adam reported,

Also, the new lesson plan [format] is time-consuming, and I do not stick to it because it is a waste of time. I do not know how much sense it makes when all that you have to do for lesson planning is to copy what you have in the teacher's edition book.

#### 8.1.4 Differentiated instruction

Over half of these interviewed teachers asserted that developing new teaching strategies that account for students' learning styles is a stressful task that requires more time than expected. Teachers explained that the lack of time limits the implementation of differentiated instruction in the classroom because they are afraid to fall behind in the action plan. Speaking about this issue, Ayman mentioned, "Honestly, I do not have time to apply numerous teaching styles that account for every student's needs every time I enter a class. If I do that, I will fall behind in the action plan." Similarly, Yomna stated,

The most stressful task is including differentiated instruction in my lesson plans and applying those in class. This is time-consuming, and honestly, I do not have enough time to apply differentiated instruction every time. If I have five students with different learning abilities or styles, it means that each session should be more than 45 minutes and I do not have more than four sessions every week.

Finally, it can be easily argued that these required areas of change are expected for most schools and teachers going through educational reform. Nevertheless, these are the concerns raised by the participating teachers, and a need for support seems timely and essential. In what follows, we summarize the reported forms of social support teachers need to overcome job-related stressors and implement change at work.

### 8.2 Emotional support: empathy empowers teachers and commands change

It is clear from teachers' responses that there is a need for various emotional support during educational change. In support of this argument, Omar stated, "The school administration is required to raise teachers' spirits and empower them to change." Teachers expressed an emotional need to feel ownership where their voices are heard and impact how change is implemented. According to Adam, "The support that teachers need at schools is being heard. The school administration should listen to us and provide us with what we need." Similarly, Rana indicated, "[Teachers] need to be heard. Without acknowledging teachers' concerns and preparing programs and plans that address them. We will not be able to implement change." These teachers argued that they could not effectively implement these changes without acknowledging teachers' concerns, coupled with the top-down type of change that directly impacts their professional lives. More importantly, teachers indicated they must believe in the viability of the reform and see leadership commitment towards reform and change.

Emotional support is needed regarding the climate of the school. Three teachers argued that not only do their views need to be acknowledged, but the school's environment should also enable them to freely express themselves and allow for creativity instead of following a set of one-size-fits-all guidelines. Speaking about the importance of autonomy and organizational democracy, Mira stated, "I would give teachers what they need. For example, more freedom to do what they find best for their students and what fits into their teaching schedule." Adam further explained, "[Teachers] need to be able to express themselves freely… If they propose a change plan, it should be taken seriously, and their creativity should be valued and encouraged." These quotes from participants demonstrate their need for a climate where they feel secure to express and have their voices heard and their suggestions used when appropriate.

Twelve teachers indicated that their emotional needs must be valued, and teachers need to be respected as teachers and for their work. Mariam commented, "I need to feel that my work is appreciated by my administration. Teachers over here work a lot, but no one acknowledges their hard work." When teachers integrate technology or work to improve their teaching, they should be valued for their improvement and commitment to work. Similarly, Farah stated, "We work a lot, and we feel underappreciated." Regarding emotional support, Sami mentioned, "The best way to motivate them, I think, is to value their hard work and show them that their seniors and supervisors are implementing change." Marwa further stated, "We need support on the psychological level. Teachers' hard work should be valued. I do not care about financial benefits. I care about someone thanking me and smiling at me at the end of the day." Finally, Samer concluded, "Teachers need to be acknowledged, appreciated, and valued because the stress that we endure is substantial." Although there are several areas of emotional support needed for these teachers in this particular setting, one could argue that many of these emotional needs would be essential in any school setting facing change.

### 8.3 Informational support: peer support bridges the knowledge gap

Regarding House (1981) category of informational support, these teachers indicated that the informational support from peers was much needed for educational changes, and peer support emerged as the dominant theme. Multiple themes emerged when teachers reported the informational support they needed to implement educational change.

Twelve teachers indicated peer support as one of the effective strategies that help them implement educational change. Speaking in this regard, Rana suggested, "I guess teachers at this school should help each other because what you know are something I do not and the opposite." Likewise, Sami stated, "some colleagues helped me out understand what I should do, but still I am not sure that I am doing it all right." Furthermore, teachers mentioned that suggestions from subject coordinators would also be a sound source of informational support. Equally important, Ayman stated, "I need peer support to learn how I apply these changes." Another teacher explained the importance of leadership support when implementing educational change. Specifically, Samar mentioned, "This is very new to us, so we are doing it on the spot, and it was not gradual. Gradually walking the teachers through it instead of introducing these changes would be more helpful."

The essence of informational support lies in the "need to know" and "be informed." Teachers stressed the need for information to meet the MEHE requirements. They need to know how to achieve goals, often needing "a step-by-step explanation" and information about why they were required to accomplish specific tasks. These teachers were open to advice and guidance from subject coordinators, colleagues, and administrators to effectively implement educational change, which will help them be more effective inside the classroom.

### 8.4 Instrumental support: accommodating teachers’ needs is a change recipe

Instrumental support centers on more direct assistance for teachers. These teachers raised the concern for the necessity of professional development opportunities and technical support for equipment in the classroom and the use of technology. Since this is a requirement from the MEHE, teachers argued they need direct support using new technology.

Seventeen teachers stressed the importance of professional development opportunities if schools are to aid in the implementation of educational change. The majority of the teachers indicated a need for professional development programs that teach them how to implement these changes and provide them with the skills needed for effective implementation of change. Speaking about the importance of professional development, Daniel stated,

I believe that teachers need training and development. They should be provided with opportunities to practice what needs to be applied before they bring change to their classrooms… To make change happen at schools, school leaders should provide professional development opportunities that focus on improving teachers' areas of weakness.

Similarly, Rasha stated, "professional development programs are essential if the school is to make these changes happen."

Ten teachers indicated that demanded changes appear to be unrealistic due to the lack of resources. Salam reported his experience with the lack of equipment and stated, "I received a Wi-Fi device and a portable projector to use in the classes that are not equipped with technology." Similarly, Mirna explained,

The most challenging and stressful part was getting a projector. I asked my coordinator for one, and he said that the department has two, and I have to share with other teachers. So, limited technology resources forced me to buy my own one and use it inside my classroom.

Finally, Samer recommended that the school should “install computer hardware and Internet connections inside each classroom to facilitate the integration of technology into instruction.”

Five teachers perceived technical support among the strategies that help schools implement educational change. Specifically, Alia explained, "Teachers at our school need technical support. We need someone who can help us overcome technical difficulties." Likewise, Samar argued, "Other than resources and time, teachers need institutional, technical support from the school. We need a team that is dedicated to helping us out when we apply these changes."

Six teachers argued for more institutional support when implementing top-down educational changes. Yomna suggested, "The most important thing is the school's ability to provide teachers with the resources they need to implement change. The school's administration's ability to adjust their structures, practices, and plans is key for successful reform implementation." Equally important, Mira further stated,

I think they [teachers] need the support of their direct boss, coordinators, and the school. I would give teachers basically more freedom in lesson planning and time management instead of handing teachers an action plan to follow. I would keep things at their own discretion and let them make decisions on their own regarding what they will explain and the time they need to spend on each lesson… Bosses should macro manage not micromanage.

These statements draw attention to the importance of leadership that is aware of teachers’ needs and takes responsibility to meet those needs in times of educational change.

### 8.5 Appraisal support: assessment transforms teaching and facilitates change

The appraisal support needed by the participating teachers centered on receiving “feedback on their work.” According to the participants, feedback is necessary to ensure that teachers are doing it right, and feedback helps them improve their practices. Six of the teachers explained that institutional research aids school administrators in tailoring professional development programs that focus on developing teachers’ skills and competencies. In support of this argument, Daniel suggested, “To make change happen at schools, school leaders should learn more about what each teacher is willing to work on and prepare professional development programs that focus on improving their areas of weakness.” Salam further stated, “The school administration should gather information to know what teachers need to make these changes a big success.”

Four teachers indicated that evaluation helps them adjust to changes and improve their performance during educational reform. According to Sarah, "[Teachers] need to be trained and should be evaluated against the required standards. They need to know if what they are doing is correct or not." Equally important, Marwa stated, "The administration should evaluate teachers based on their qualifications, experience, and records of success." Tania explained, "Teachers need sufficient resources and constant evaluation and feedback from their subject coordinators. Student feedback is also important." Finally, six teachers concluded that feedback aids them in determining whether their performance and practices are effective and valuable. According to Jaida, "Teachers should receive feedback from their students and subject coordinators to know whether they are correct and beneficial." Farah further explained, "Teachers need someone who assists them in applying these changes and provides them with feedback to help them improve their practices."

## 9 Discussion

Regarding the job stresses that obstruct the implementation of the reform, this study found that teachers face job stresses that primarily centered on the need to improve or change their teaching practices. More specifically, they face challenges such as integrating technology into their teaching, redesigning curriculum, new regulations regarding lesson plan formatting, and developing and implementing differentiated instruction. These findings align with previous research on teachers in Qatar's government schools. During the reform, teachers in Qatar's government schools had to adapt their teaching practices, including integrating technology and considering student learning (Romanowski et al., 2013). Like government teachers, private school teachers in this study reported that the reform impacted their planning (Alfadala, 2015; Romanowski et al., 2013). Teachers in Qatar were also involved in the development of curriculum design (Stasz et al., 2007).

In this study, teachers highlighted the importance of emotional support suggesting that they lacked a voice in the reform. They felt underappreciated and that their ideas regarding implementation were not valued, seldom thanked, and never felt the administration value their work. This finding aligns with Romanowski et al. (2013) and Mullick (2013). They reported that in education reforms in Qatar and Saudi Arabia, teachers' voices are often absent in the development and implementation of top-down educational reforms leading to feeling unappreciated by educational policymakers and leaders. Scott (2019) found that teachers who do not receive adequate emotional support from colleagues and principals doubt their abilities.

Regarding informational support, teachers in this study indicated a need to receive informational support from colleagues and school administrators to implement the various changes better. The need for peer support through providing and sharing information is necessary for these teachers. Teachers indicated they lacked information about the reform or "how-to" information or assistance that walked them through the proper way to implement change. They often felt like they were in the dark or not fully informed and needed guidance and details from leaders and peers. This lack of information hampers the implementation of various required changes. Greenglass et al. (1997) found that the support provided by colleagues contained informational, practical, and emotional aspects, and this knowledge enables teachers to believe they had more control over their jobs.

Studies conducted in UAE, a culturally similar context to Qatar on teacher resistance, found that teachers resist change due to governmental interference and imposition, fear of the unknown, language barriers, and that government mandates overwhelm teachers and degrade their work conditions (Ibrahim et al., 2013; Tabari, 2014). Specifically in Qatar, Alfadala (2015) examined the reform efforts over the past 15 years in Qatar, the United Arab Emirates (UAE), and the Kingdom of Saudi Arabia (KSA) and found the challenges facing educational reform in these countries are primarily caused by teachers’ resistance to change.

These teachers also raised the need for instrumental support such as professional development that addresses their specific needs, such as using technology. They also argued that school leadership should provide the resources and opportunities to develop the skills needed to implement the required changes. Teachers in this study stressed the importance of training programs, on-time technical support, ample time, and senior leadership commitment when implementing educational change. Also, they requested more time so they could more effectively implement changes. Mounting evidence suggests that educational reform necessitates institutional and technical support (An and Reigeluth, 2014; Vrasidas, 2014), professional development (Girvan et al., 2016), and sufficient allocation of resources (Johnson et al., 2012). The findings from this study align with similar studies that examined the effects of instrumental support on teachers' performance and contribution to change during educational reform. For example, Lam et al. (2010) found that teachers are more likely to change and implement reform in a collegial school culture that emphasizes autonomy and responds to teachers' needs by reducing their workload and providing them with needed financial and material resources.

Finally, teachers reported a need for appraisal support through in feedback so they know if they are implementing change correctly. They also expressed a need for professional development to develop the specific skills needed to implement change. This requires that leaders collect data to identify the skills teachers need to be able to implement change. The essence of appraisal support for these teachers is knowing whether they are applying changes correctly. The feedback they receive from their colleagues should add to their self-efficacy as teachers. These results are consistent with the literature related to appraisal support (Karkouti et al., 2021; Samuel and Berhanu, 2019) and support the work of Wright (2017), who found that the lack of appraisal support deteriorates teacher-principal relationships, increases stress in the workplace, and obstructs the implementation of educational change.

## 10 Recommendations

Findings from this study will help Qatari educational leaders understand the effects of social and professional support on teachers' willingness and acceptance to change. This information can be of primary importance for Qatari policymakers as they develop strategies that propel change and support the implementation of the current reform.

Principals play a crucial role in education reform and can facilitate or hinder change. However, principals are less likely to lead change in schools in developing countries and simply follow the bureaucratic values of stability and routine (Oplatka, 2018). Therefore, educational leaders must create an awareness of how educational reform impacts teachers personally and professionally and provide appropriate types of social support that will aid teachers in coping effectively with the many changes. In turn, this influences the fidelity of implementation, which determines how well a program is implemented compared to the original program design (O'Donnell, 2008).

Principals should provide emotional support that demonstrates that teachers are appreciated as they implement change. More importantly, especially in top-down reforms, they need to establish trusting relationships with teachers. Balyer (2017) suggests that trust is a resource for principals implementing improvement plans. Also, providing accurate information that keeps teachers informed about the purpose of changes will facilitate the process. More importantly, there needs to be an avenue where teachers’ questions and concerns can be accurately addressed. Instrumental support is valuable and can provide the required time for teachers to deal with change and develop the skills required to implement change. Reduction in non-teaching duties will give the time that is necessary to implement change effectively. Finally, appraisal support provides teachers with feedback that enables them to develop self-efficacy as they implement change.

Social support should not be the sole responsibility of principals, but colleagues and co-workers should be a source of support as well. Thus, principals should cultivate a climate where there is an awareness of others and their needs for social support and possibly utilize peer support teams within schools. Also, schools must provide an atmosphere that allows teachers to feel safe as they implement change and secure as they seek advice, guidance, or other needed forms of social support.

Policymakers do not necessarily pay much attention to the implementation of reform and their policies. Customarily, policymakers consider education policy implementation as the practical stage of the process where their decision is implemented by administrators and educators (Viennet and Pont, 2017). Since policymakers are the initiators of reform and change, they too should establish an awareness of how reform and the policies they develop will impact the lives of teachers. It would be helpful that policymakers provide a framework designed to support educators in the implementation process. The framework should include elements of social support that can contribute to success in the policy process.

In closing, this study improves the understanding of social and professional support needed by teachers as they implement educational change. As Qatar continues to implement educational change and reform, policymakers and principals in Qatar must respond to teachers' needs and actively engage them in the decision-making process to realize QNSA's vision and implement lasting change in education.

## Declarations

### Author contribution statement

Michael H. Romanowski: Analyzed and interpreted the data; Wrote the paper.

Reem Khalid Abu- Shawish:Analyzed and interpreted the data; Wrote the paper.

Ibrahim M. Karkouti:Conceived and designed the experiments; Performed the experiments; Analyzed and interpreted the data; Wrote the paper.

### Funding statement

This research did not receive any specific grant from funding agencies in the public, commercial, or not-for-profit sectors.

### Data availability statement

The data that has been used is confidential.

### Declaration of interests statement

The authors declare no conflict of interest.

### Additional information

No additional information is available for this paper.

## Appendix A

### **Interview Protocol**

1. What are the most difficult or stressful changes required by the Ministry of Education and Higher Education you have faced at this school? May you please provide an example?
2. If you were in charge of implementing these changes, what would you do to help teachers? Any example?
3. What types of support do teachers need when they deal with educational changes?
4. What type of support did you receive as you implemented these changes? From whom? Were they helpful? May you please provide an example?
5. Is providing support a one-size-fits all model or do individual teachers need different types of support? Any example?
6. When speaking about support, what do you think teachers need the most to make change happen at schools?

## Footnotes

1 The pseudonym, New International School or NIS, will be used as the school's name, where this study was conducted throughout this paper.
